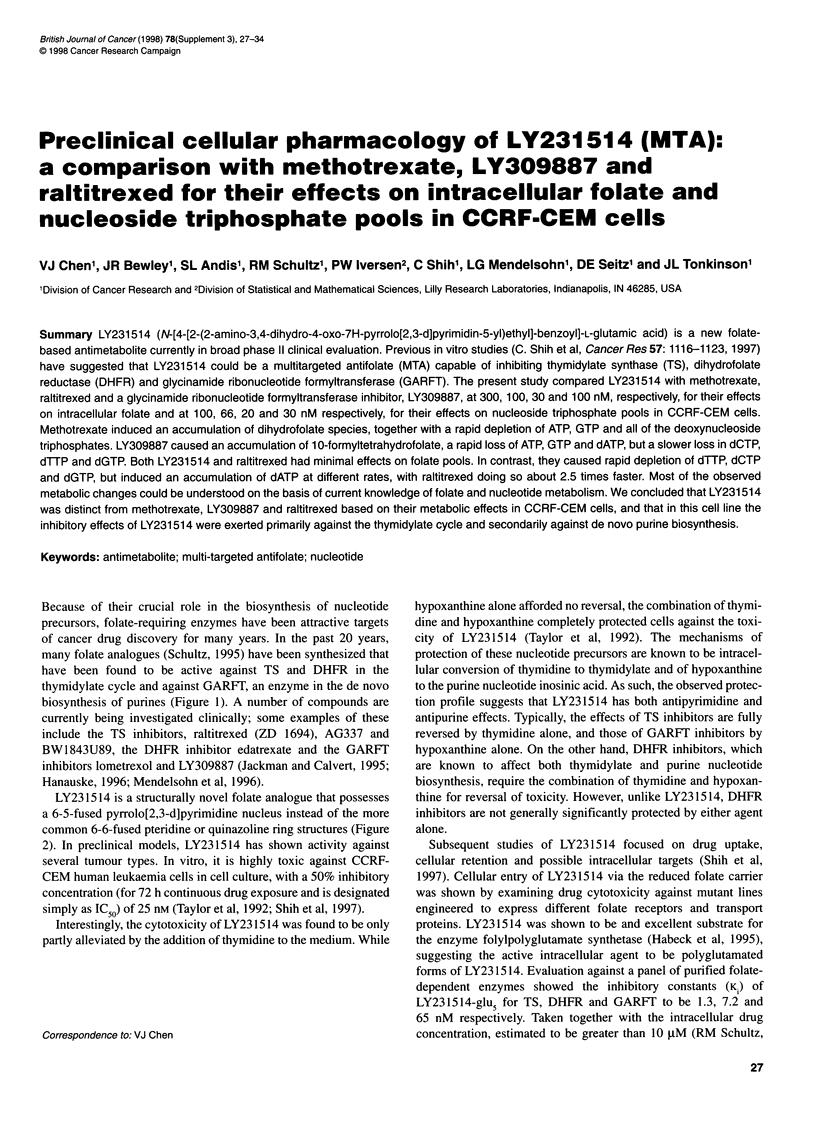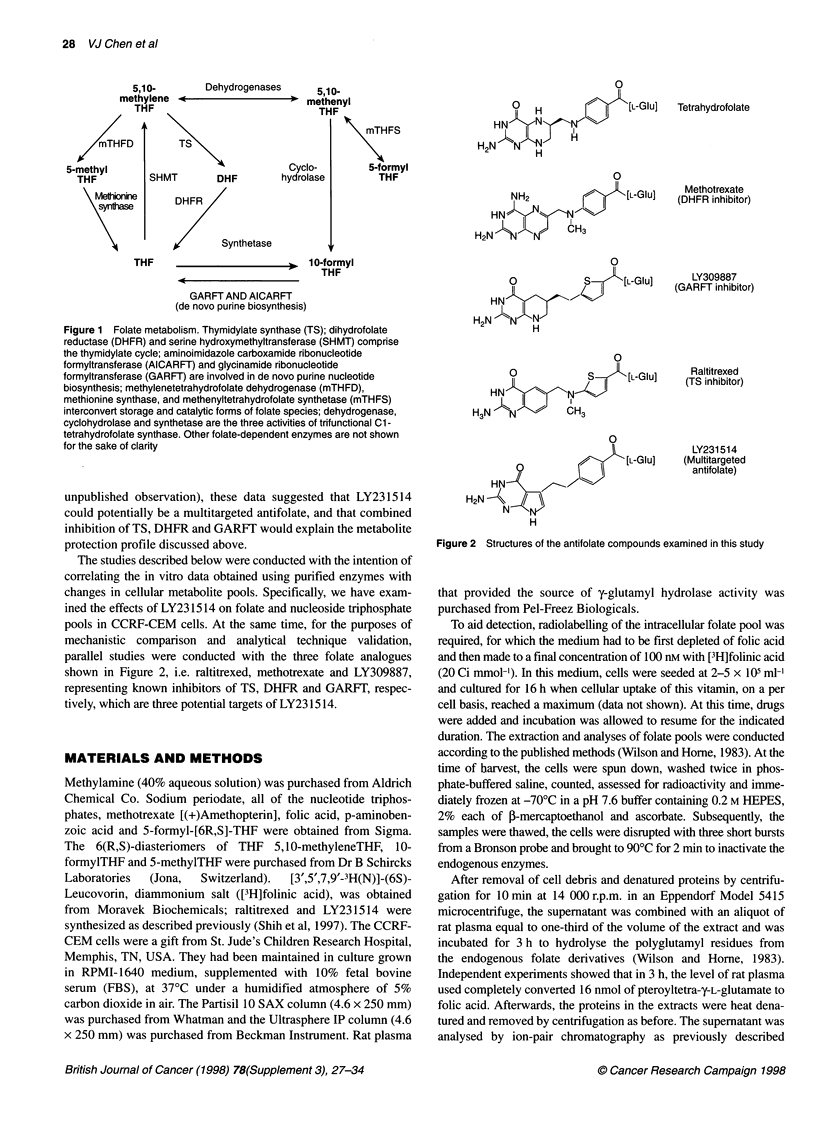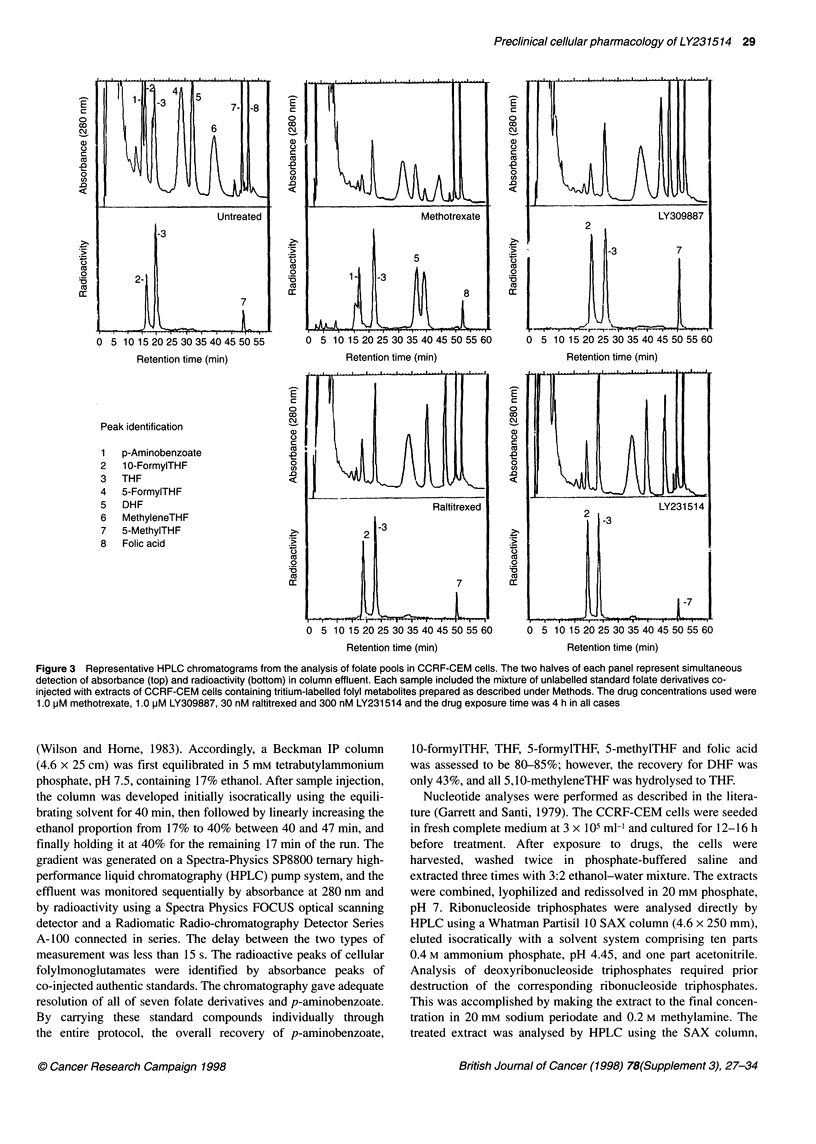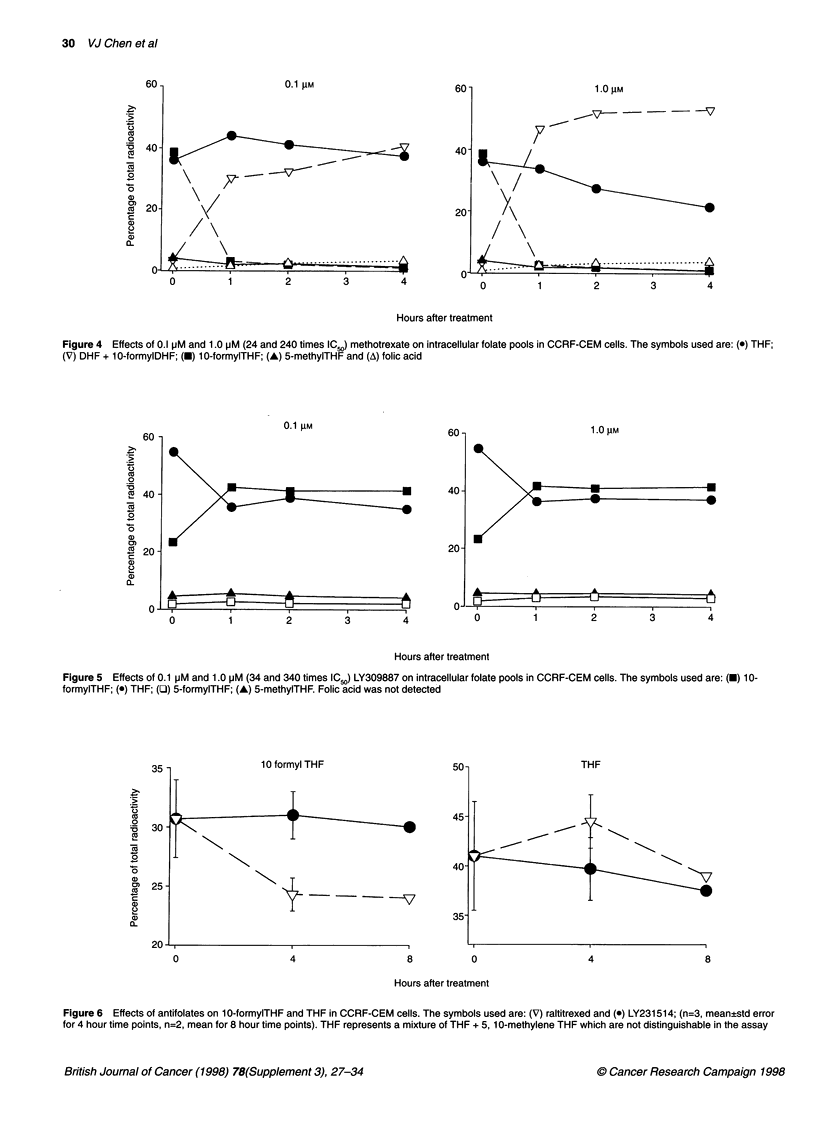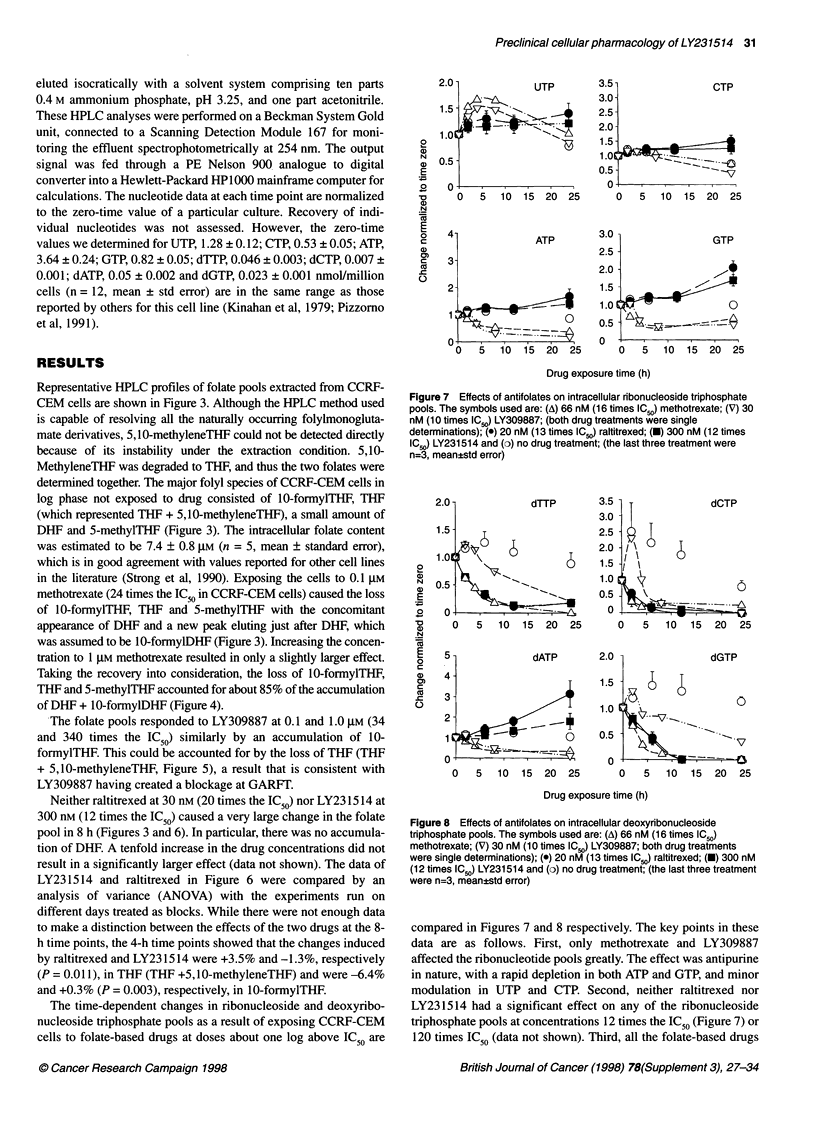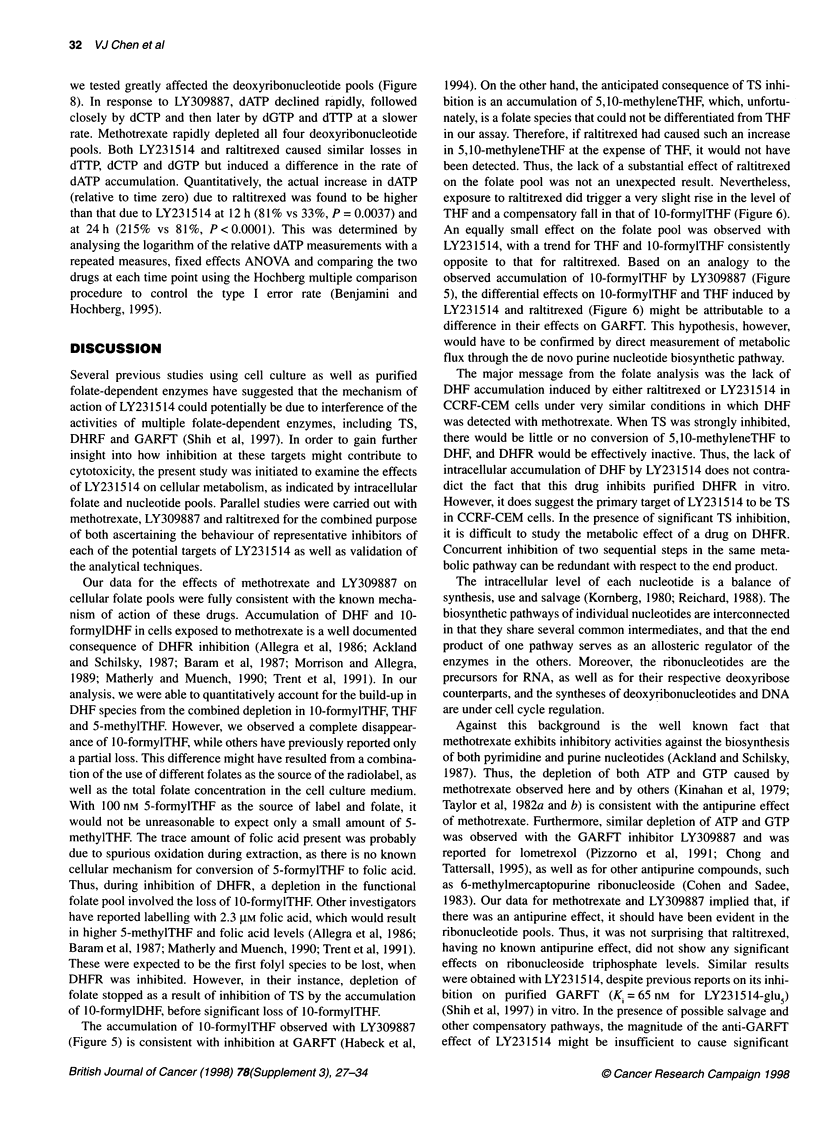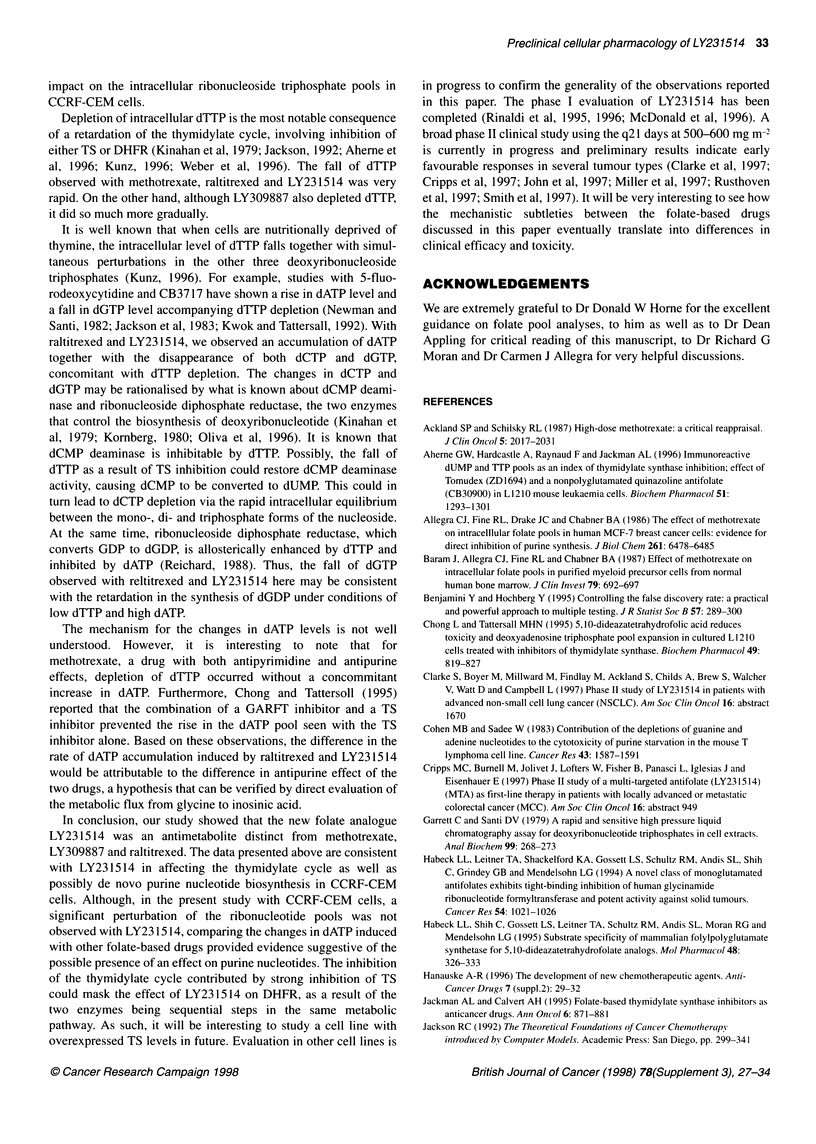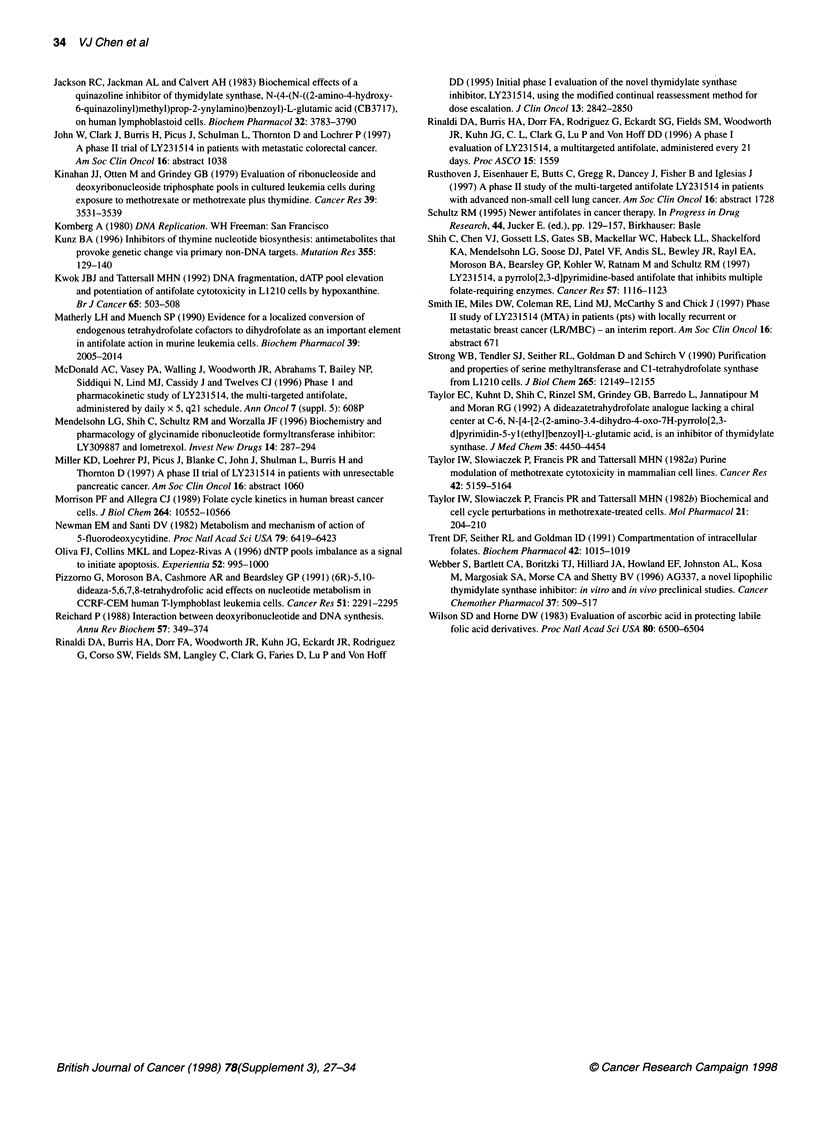# Preclinical cellular pharmacology of LY231514 (MTA): a comparison with methotrexate, LY309887 and raltitrexed for their effects on intracellular folate and nucleoside triphosphate pools in CCRF-CEM cells.

**DOI:** 10.1038/bjc.1998.751

**Published:** 1998

**Authors:** V. J. Chen, J. R. Bewley, S. L. Andis, R. M. Schultz, P. W. Iversen, C. Shih, L. G. Mendelsohn, D. E. Seitz, J. L. Tonkinson

**Affiliations:** Division of Cancer Research, Lilly Research Laboratories, Indianapolis, IN 46285, USA.

## Abstract

LY231514 (N-[4-[2-(2-amino-3,4-dihydro-4-oxo-7H-pyrrolo[2,3-d]pyrimidin-5-yl)ethy l]-benzoyl]-L-glutamic acid) is a new folate-based antimetabolite currently in broad phase II clinical evaluation. Previous in vitro studies (C. Shih et al, CancerRes 57: 1116-1123, 1997) have suggested that LY231514 could be a multitargeted antifolate (MTA) capable of inhibiting thymidylate synthase (TS), dihydrofolate reductase (DHFR) and glycinamide ribonucleotide formyltransferase (GARFT). The present study compared LY231514 with methotrexate, raltitrexed and a glycinamide ribonucleotide formyltransferase inhibitor, LY309887, at 300, 100, 30 and 100 nM, respectively, for their effects on intracellular folate and at 100, 66, 20 and 30 nM respectively, for their effects on nucleoside triphosphate pools in CCRF-CEM cells. Methotrexate induced an accumulation of dihydrofolate species, together with a rapid depletion of ATP, GTP and all of the deoxynucleoside triphosphates. LY309887 caused an accumulation of 10-formyltetrahydrofolate, a rapid loss of ATP, GTP and dATP, but a slower loss in dCTP, dTTP and dGTP. Both LY231514 and raltitrexed had minimal effects on folate pools. In contrast, they caused rapid depletion of dTTP, dCTP and dGTP, but induced an accumulation of dATP at different rates, with raltitrexed doing so about 2.5 times faster. Most of the observed metabolic changes could be understood on the basis of current knowledge of folate and nucleotide metabolism. We concluded that LY231514 was distinct from methotrexate, LY309887 and raltitrexed based on their metabolic effects in CCRF-CEM cells, and that in this cell line the inhibitory effects of LY231514 were exerted primarily against the thymidylate cycle and secondarily against de novo purine biosynthesis.


British Journal of Cancer (1998) 78(Supplement 3), 27–34
© 1998 Cancer Research Campaign

### Preclinical cellular pharmacology of LY231514 (MTA): a comparison with methotrexate, LY309887 and raltitrexed for their effects on intracellular folate and nucleoside triphosphate pools in CCRF-CEM cells

**VJ Chen[1], JR Bewley[1], SL Andis[1], RM Schultz[1], PW Iversen[2], C Shih[1], LG Mendelsohn[1], DE Seitz[1] and JL Tonkinson[1]**

[1]Division of Cancer Research and [2]Division of Statistical and Mathematical Sciences, Lilly Research Laboratories, Indianapolis, IN 46285, USA

**Summary** LY231514 (*N*-[4-[2-(2-amino-3,4-dihydro-4-oxo-7H-pyrrolo[2,3-d]pyrimidin-5-yl)ethyl]-benzoyl]-L-glutamic acid) is a new folate-based antimetabolite currently in broad phase II clinical evaluation. Previous in vitro studies (C. Shih et al, *Cancer Res* **57**: 1116–1123, 1997) have suggested that LY231514 could be a multitargeted antifolate (MTA) capable of inhibiting thymidylate synthase (TS), dihydrofolate reductase (DHFR) and glycinamide ribonucleotide formyltransferase (GARFT). The present study compared LY231514 with methotrexate, raltitrexed and a glycinamide ribonucleotide formyltransferase inhibitor, LY309887, at 300, 100, 30 and 100 nM, respectively, for their effects on intracellular folate and at 100, 66, 20 and 30 nM respectively, for their effects on nucleoside triphosphate pools in CCRF-CEM cells. Methotrexate induced an accumulation of dihydrofolate species, together with a rapid depletion of ATP, GTP and all of the deoxynucleoside triphosphates. LY309887 caused an accumulation of 10-formyltetrahydrofolate, a rapid loss of ATP, GTP and dATP, but a slower loss in dCTP, dTTP and dGTP. Both LY231514 and raltitrexed had minimal effects on folate pools. In contrast, they caused rapid depletion of dTTP, dCTP and dGTP, but induced an accumulation of dATP at different rates, with raltitrexed doing so about 2.5 times faster. Most of the observed metabolic changes could be understood on the basis of current knowledge of folate and nucleotide metabolism. We concluded that LY231514 was distinct from methotrexate, LY309887 and raltitrexed based on their metabolic effects in CCRF-CEM cells, and that in this cell line the inhibitory effects of LY231514 were exerted primarily against the thymidylate cycle and secondarily against de novo purine biosynthesis.

**Keywords:** antimetabolite; multi-targeted antifolate; nucleotide

Because of their crucial role in the biosynthesis of nucleotide precursors, folate-requiring enzymes have been attractive targets of cancer drug discovery for many years. In the past 20 years, many folate analogues (Schultz, 1995) have been synthesized that have been found to be active against TS and DHFR in the thymidylate cycle and against GARFT, an enzyme in the de novo biosynthesis of purines (Figure 1). A number of compounds are currently being investigated clinically; some examples of these include the TS inhibitors, raltitrexed (ZD 1694), AG337 and BW1843U89, the DHFR inhibitor edatrexate and the GARFT inhibitors lometrexol and LY309887 (Jackman and Calvert, 1995; Hanauske, 1996; Mendelsohn et al, 1996).

LY231514 is a structurally novel folate analogue that possesses a 6-5-fused pyrrolo[2,3-d]pyrimidine nucleus instead of the more common 6-6-fused pteridine or quinazoline ring structures (Figure 2). In preclinical models, LY231514 has shown activity against several tumour types. In vitro, it is highly toxic against CCRF-CEM human leukaemia cells in cell culture, with a 50% inhibitory concentration (for 72 h continuous drug exposure and is designated simply as $IC_{50}$) of 25 nM (Taylor et al, 1992; Shih et al, 1997).

Interestingly, the cytotoxicity of LY231514 was found to be only partly alleviated by the addition of thymidine to the medium. While hypoxanthine alone afforded no reversal, the combination of thymidine and hypoxanthine completely protected cells against the toxicity of LY231514 (Taylor et al, 1992). The mechanisms of protection of these nucleotide precursors are known to be intracellular conversion of thymidine to thymidylate and of hypoxanthine to the purine nucleotide inosinic acid. As such, the observed protection profile suggests that LY231514 has both antipyrimidine and antipurine effects. Typically, the effects of TS inhibitors are fully reversed by thymidine alone, and those of GARFT inhibitors by hypoxanthine alone. On the other hand, DHFR inhibitors, which are known to affect both thymidylate and purine nucleotide biosynthesis, require the combination of thymidine and hypoxanthine for reversal of toxicity. However, unlike LY231514, DHFR inhibitors are not generally significantly protected by either agent alone.

Subsequent studies of LY231514 focused on drug uptake, cellular retention and possible intracellular targets (Shih et al, 1997). Cellular entry of LY231514 via the reduced folate carrier was shown by examining drug cytotoxicity against mutant lines engineered to express different folate receptors and transport proteins. LY231514 was shown to be and excellent substrate for the enzyme folylpolyglutamate synthetase (Habeck et al, 1995), suggesting the active intracellular agent to be polyglutamated forms of LY231514. Evaluation against a panel of purified folate-dependent enzymes showed the inhibitory constants ($K_i$) of LY231514-glu$_5$ for TS, DHFR and GARFT to be 1.3, 7.2 and 65 nM respectively. Taken together with the intracellular drug concentration, estimated to be greater than 10 μM (RM Schultz,

*Correspondence to:* VJ Chen

**Figure 1** Folate metabolism. Thymidylate synthase (TS); dihydrofolate reductase (DHFR) and serine hydroxymethyltransferase (SHMT) comprise the thymidylate cycle; aminoimidazole carboxamide ribonucleotide formyltransferase (AICARFT) and glycinamide ribonucleotide formyltransferase (GARFT) are involved in de novo purine nucleotide biosynthesis; methylenetetrahydrofolate dehydrogenase (mTHFD), methionine synthase, and methenyltetrahydrofolate synthetase (mTHFS) interconvert storage and catalytic forms of folate species; dehydrogenase, cyclohydrolase and synthetase are the three activities of trifunctional C1-tetrahydrofolate synthase. Other folate-dependent enzymes are not shown for the sake of clarity

**Figure 2** Structures of the antifolate compounds examined in this study unpublished observation), these data suggested that LY231514 could potentially be a multitargeted antifolate, and that combined inhibition of TS, DHFR and GARFT would explain the metabolite protection profile discussed above.

The studies described below were conducted with the intention of correlating the in vitro data obtained using purified enzymes with changes in cellular metabolite pools. Specifically, we have examined the effects of LY231514 on folate and nucleoside triphosphate pools in CCRF-CEM cells. At the same time, for the purposes of mechanistic comparison and analytical technique validation, parallel studies were conducted with the three folate analogues shown in Figure 2, i.e. raltitrexed, methotrexate and LY309887, representing known inhibitors of TS, DHFR and GARFT, respectively, which are three potential targets of LY231514.

### MATERIALS AND METHODS

Methylamine (40% aqueous solution) was purchased from Aldrich Chemical Co. Sodium periodate, all of the nucleotide triphosphates, methotrexate [(+)Amethopterin], folic acid, p-aminobenzoic acid and 5-formyl-[6R,S]-THF were obtained from Sigma. The 6(R,S)-diasteriomers of THF 5,10-methyleneTHF, 10-formylTHF and 5-methylTHF were purchased from Dr B Schircks Laboratories (Jona, Switzerland). [3′,5′,7,9′-$^{3}$H(N)]-(6S)-Leucovorin, diammonium salt ([$^{3}$H]folinic acid), was obtained from Moravek Biochemicals; raltitrexed and LY231514 were synthesized as described previously (Shih et al, 1997). The CCRF-CEM cells were a gift from St. Jude's Children Research Hospital, Memphis, TN, USA. They had been maintained in culture grown in RPMI-1640 medium, supplemented with 10% fetal bovine serum (FBS), at 37°C under a humidified atmosphere of 5% carbon dioxide in air. The Partisil 10 SAX column (4.6 × 250 mm) was purchased from Whatman and the Ultrasphere IP column (4.6 × 250 mm) was purchased from Beckman Instrument. Rat plasma that provided the source of γ-glutamyl hydrolase activity was purchased from Pel-Freez Biologicals.

To aid detection, radiolabelling of the intracellular folate pool was required, for which the medium had to be first depleted of folic acid and then made to a final concentration of 100 nM with [$^{3}$H]folinic acid (20 Ci mmol$^{-1}$). In this medium, cells were seeded at 2–5 × $10^{5}$ ml$^{-1}$ and cultured for 16 h when cellular uptake of this vitamin, on a per cell basis, reached a maximum (data not shown). At this time, drugs were added and incubation was allowed to resume for the indicated duration. The extraction and analyses of folate pools were conducted according to the published methods (Wilson and Horne, 1983). At the time of harvest, the cells were spun down, washed twice in phosphate-buffered saline, counted, assessed for radioactivity and immediately frozen at –70°C in a pH 7.6 buffer containing 0.2 M HEPES, 2% each of β-mercaptoethanol and ascorbate. Subsequently, the samples were thawed, the cells were disrupted with three short bursts from a Bronson probe and brought to 90°C for 2 min to inactivate the endogenous enzymes.

After removal of cell debris and denatured proteins by centrifugation for 10 min at 14 000 r.p.m. in an Eppendorf Model 5415 microcentrifuge, the supernatant was combined with an aliquot of rat plasma equal to one-third of the volume of the extract and was incubated for 3 h to hydrolyse the polyglutamyl residues from the endogenous folate derivatives (Wilson and Horne, 1983). Independent experiments showed that in 3 h, the level of rat plasma used completely converted 16 nmol of pteroyltetra-γ-L-glutamate to folic acid. Afterwards, the proteins in the extracts were heat denatured and removed by centrifugation as before. The supernatant was analysed by ion-pair chromatography as previously described

© Cancer Research Campaign 1998

**Figure 3** Representative HPLC chromatograms from the analysis of folate pools in CCRF-CEM cells. The two halves of each panel represent simultaneous detection of absorbance (top) and radioactivity (bottom) in column effluent. Each sample included the mixture of unlabelled standard folate derivatives co-injected with extracts of CCRF-CEM cells containing tritium-labelled folyl metabolites prepared as described under Methods. The drug concentrations used were 1.0 μM methotrexate, 1.0 μM LY309887, 30 nM raltitrexed and 300 nM LY231514 and the drug exposure time was 4 h in all cases

(Wilson and Horne, 1983). Accordingly, a Beckman IP column (4.6 × 25 cm) was first equilibrated in 5 mM tetrabutylammonium phosphate, pH 7.5, containing 17% ethanol. After sample injection, the column was developed initially isocratically using the equilibrating solvent for 40 min, then followed by linearly increasing the ethanol proportion from 17% to 40% between 40 and 47 min, and finally holding it at 40% for the remaining 17 min of the run. The gradient was generated on a Spectra-Physics SP8800 ternary high-performance liquid chromatography (HPLC) pump system, and the effluent was monitored sequentially by absorbance at 280 nm and by radioactivity using a Spectra Physics FOCUS optical scanning detector and a Radiomatic Radio-chromatography Detector Series A-100 connected in series. The delay between the two types of measurement was less than 15 s. The radioactive peaks of cellular folylmonoglutamates were identified by absorbance peaks of co-injected authentic standards. The chromatography gave adequate resolution of all of seven folate derivatives and *p*-aminobenzoate. By carrying these standard compounds individually through the entire protocol, the overall recovery of *p*-aminobenzoate, 10-formylTHF, THF, 5-formylTHF, 5-methylTHF and folic acid was assessed to be 80–85%; however, the recovery for DHF was only 43%, and all 5,10-methyleneTHF was hydrolysed to THF.

Nucleotide analyses were performed as described in the literature (Garrett and Santi, 1979). The CCRF-CEM cells were seeded in fresh complete medium at $3 \times 10^5$ $ml^{-1}$ and cultured for 12–16 h before treatment. After exposure to drugs, the cells were harvested, washed twice in phosphate-buffered saline and extracted three times with 3:2 ethanol–water mixture. The extracts were combined, lyophilized and redissolved in 20 mM phosphate, pH 7. Ribonucleoside triphosphates were analysed directly by HPLC using a Whatman Partisil 10 SAX column (4.6 × 250 mm), eluted isocratically with a solvent system comprising ten parts 0.4 M ammonium phosphate, pH 4.45, and one part acetonitrile. Analysis of deoxyribonucleoside triphosphates required prior destruction of the corresponding ribonucleoside triphosphates. This was accomplished by making the extract to the final concentration in 20 mM sodium periodate and 0.2 M methylamine. The treated extract was analysed by HPLC using the SAX column,

© *Cancer Research Campaign 1998*

**Figure 4** Effects of 0.I μM and 1.0 μM (24 and 240 times $IC_{50}$) methotrexate on intracellular folate pools in CCRF-CEM cells. The symbols used are: (•) THF; (∇) DHF + 10-formylDHF; (■) 10-formylTHF; (▲) 5-methylTHF and (Δ) folic acid

**Figure 5** Effects of 0.1 μM and 1.0 μM (34 and 340 times $IC_{50}$) LY309887 on intracellular folate pools in CCRF-CEM cells. The symbols used are: (■) 10-formylTHF; (•) THF; (□) 5-formylTHF; (▲) 5-methylTHF. Folic acid was not detected

**Figure 6** Effects of antifolates on 10-formylTHF and THF in CCRF-CEM cells. The symbols used are: (∇) raltitrexed and (•) LY231514; (n=3, mean±std error for 4 hour time points, n=2, mean for 8 hour time points). THF represents a mixture of THF + 5, 10-methylene THF which are not distinguishable in the assay

 © Cancer Research Campaign 1998

eluted isocratically with a solvent system comprising ten parts 0.4 M ammonium phosphate, pH 3.25, and one part acetonitrile. These HPLC analyses were performed on a Beckman System Gold unit, connected to a Scanning Detection Module 167 for monitoring the effluent spectrophotometrically at 254 nm. The output signal was fed through a PE Nelson 900 analogue to digital converter into a Hewlett-Packard HP1000 mainframe computer for calculations. The nucleotide data at each time point are normalized to the zero-time value of a particular culture. Recovery of individual nucleotides was not assessed. However, the zero-time values we determined for UTP, 1.28 ± 0.12; CTP, 0.53 ± 0.05; ATP, 3.64 ± 0.24; GTP, 0.82 ± 0.05; dTTP, 0.046 ± 0.003; dCTP, 0.007 ± 0.001; dATP, 0.05 ± 0.002 and dGTP, 0.023 ± 0.001 nmol/million cells (n = 12, mean ± std error) are in the same range as those reported by others for this cell line (Kinahan et al, 1979; Pizzorno et al, 1991).

### RESULTS

Representative HPLC profiles of folate pools extracted from CCRF-CEM cells are shown in Figure 3. Although the HPLC method used is capable of resolving all the naturally occurring folylmonoglutamate derivatives, 5,10-methyleneTHF could not be detected directly because of its instability under the extraction condition. 5,10-MethyleneTHF was degraded to THF, and thus the two folates were determined together. The major folyl species of CCRF-CEM cells in log phase not exposed to drug consisted of 10-formylTHF, THF (which represented THF + 5,10-methyleneTHF), a small amount of DHF and 5-methylTHF (Figure 3). The intracellular folate content was estimated to be 7.4 ± 0.8 μM (*n* = 5, mean ± standard error), which is in good agreement with values reported for other cell lines in the literature (Strong et al, 1990). Exposing the cells to 0.1 μM methotrexate (24 times the $IC_{50}$ in CCRF-CEM cells) caused the loss of 10-formylTHF, THF and 5-methylTHF with the concomitant appearance of DHF and a new peak eluting just after DHF, which was assumed to be 10-formylDHF (Figure 3). Increasing the concentration to 1 μM methotrexate resulted in only a slightly larger effect. Taking the recovery into consideration, the loss of 10-formylTHF, THF and 5-methylTHF accounted for about 85% of the accumulation of DHF + 10-formylDHF (Figure 4).

The folate pools responded to LY309887 at 0.1 and 1.0 μM (34 and 340 times the $IC_{50}$) similarly by an accumulation of 10-formylTHF. This could be accounted for by the loss of THF (THF + 5,10-methyleneTHF, Figure 5), a result that is consistent with LY309887 having created a blockage at GARFT.

Neither raltitrexed at 30 nM (20 times the $IC_{50}$) nor LY231514 at 300 nM (12 times the $IC_{50}$) caused a very large change in the folate pool in 8 h (Figures 3 and 6). In particular, there was no accumulation of DHF. A tenfold increase in the drug concentrations did not result in a significantly larger effect (data not shown). The data of LY231514 and raltitrexed in Figure 6 were compared by an analysis of variance (ANOVA) with the experiments run on different days treated as blocks. While there were not enough data to make a distinction between the effects of the two drugs at the 8-h time points, the 4-h time points showed that the changes induced by raltitrexed and LY231514 were +3.5% and –1.3%, respectively ($P = 0.011$), in THF (THF +5,10-methyleneTHF) and were –6.4% and +0.3% ($P = 0.003$), respectively, in 10-formylTHF.

The time-dependent changes in ribonucleoside and deoxyribonucleoside triphosphate pools as a result of exposing CCRF-CEM cells to folate-based drugs at doses about one log above $IC_{50}$ are compared in Figures 7 and 8 respectively. The key points in these data are as follows. First, only methotrexate and LY309887 affected the ribonucleotide pools greatly. The effect was antipurine in nature, with a rapid depletion in both ATP and GTP, and minor modulation in UTP and CTP. Second, neither raltitrexed nor LY231514 had a significant effect on any of the ribonucleoside triphosphate pools at concentrations 12 times the $IC_{50}$ (Figure 7) or 120 times $IC_{50}$ (data not shown). Third, all the folate-based drugs

**Figure 7** Effects of antifolates on intracellular ribonucleoside triphosphate pools. The symbols used are: (Δ) 66 nM (16 times $IC_{50}$) methotrexate; (∇) 30 nM (10 times $IC_{50}$) LY309887; (both drug treatments were single determinations); (●) 20 nM (13 times $IC_{50}$) raltitrexed; (■) 300 nM (12 times $IC_{50}$) LY231514 and (○) no drug treatment; (the last three treatment were n=3, mean±std error)

**Figure 8** Effects of antifolates on intracellular deoxyribonucleoside triphosphate pools. The symbols used are: (Δ) 66 nM (16 times $IC_{50}$) methotrexate; (∇) 30 nM (10 times $IC_{50}$) LY309887; both drug treatments were single determinations); (●) 20 nM (13 times $IC_{50}$) raltitrexed; (■) 300 nM (12 times $IC_{50}$) LY231514 and (○) no drug treatment; (the last three treatment were n=3, mean±std error)

© Cancer Research Campaign 1998

we tested greatly affected the deoxyribonucleotide pools (Figure 8). In response to LY309887, dATP declined rapidly, followed closely by dCTP and then later by dGTP and dTTP at a slower rate. Methotrexate rapidly depleted all four deoxyribonucleotide pools. Both LY231514 and raltitrexed caused similar losses in dTTP, dCTP and dGTP but induced a difference in the rate of dATP accumulation. Quantitatively, the actual increase in dATP (relative to time zero) due to raltitrexed was found to be higher than that due to LY231514 at 12 h (81% vs 33%, $P = 0.0037$) and at 24 h (215% vs 81%, $P < 0.0001$). This was determined by analysing the logarithm of the relative dATP measurements with a repeated measures, fixed effects ANOVA and comparing the two drugs at each time point using the Hochberg multiple comparison procedure to control the type I error rate (Benjamini and Hochberg, 1995).

### DISCUSSION

Several previous studies using cell culture as well as purified folate-dependent enzymes have suggested that the mechanism of action of LY231514 could potentially be due to interference of the activities of multiple folate-dependent enzymes, including TS, DHRF and GARFT (Shih et al, 1997). In order to gain further insight into how inhibition at these targets might contribute to cytotoxicity, the present study was initiated to examine the effects of LY231514 on cellular metabolism, as indicated by intracellular folate and nucleotide pools. Parallel studies were carried out with methotrexate, LY309887 and raltitrexed for the combined purpose of both ascertaining the behaviour of representative inhibitors of each of the potential targets of LY231514 as well as validation of the analytical techniques.

Our data for the effects of methotrexate and LY309887 on cellular folate pools were fully consistent with the known mechanism of action of these drugs. Accumulation of DHF and 10-formylDHF in cells exposed to methotrexate is a well documented consequence of DHFR inhibition (Allegra et al, 1986; Ackland and Schilsky, 1987; Baram et al, 1987; Morrison and Allegra, 1989; Matherly and Muench, 1990; Trent et al, 1991). In our analysis, we were able to quantitatively account for the build-up in DHF species from the combined depletion in 10-formylTHF, THF and 5-methylTHF. However, we observed a complete disappearance of 10-formylTHF, while others have previously reported only a partial loss. This difference might have resulted from a combination of the use of different folates as the source of the radiolabel, as well as the total folate concentration in the cell culture medium. With 100 nM 5-formylTHF as the source of label and folate, it would not be unreasonable to expect only a small amount of 5-methylTHF. The trace amount of folic acid present was probably due to spurious oxidation during extraction, as there is no known cellular mechanism for conversion of 5-formylTHF to folic acid. Thus, during inhibition of DHFR, a depletion in the functional folate pool involved the loss of 10-formylTHF. Other investigators have reported labelling with 2.3 μM folic acid, which would result in higher 5-methylTHF and folic acid levels (Allegra et al, 1986; Baram et al, 1987; Matherly and Muench, 1990; Trent et al, 1991). These were expected to be the first folyl species to be lost, when DHFR was inhibited. However, in their instance, depletion of folate stopped as a result of inhibition of TS by the accumulation of 10-formylDHF, before significant loss of 10-formylTHF.

The accumulation of 10-formylTHF observed with LY309887 (Figure 5) is consistent with inhibition at GARFT (Habeck et al, 1994). On the other hand, the anticipated consequence of TS inhibition is an accumulation of 5,10-methyleneTHF, which, unfortunately, is a folate species that could not be differentiated from THF in our assay. Therefore, if raltitrexed had caused such an increase in 5,10-methyleneTHF at the expense of THF, it would not have been detected. Thus, the lack of a substantial effect of raltitrexed on the folate pool was not an unexpected result. Nevertheless, exposure to raltitrexed did trigger a very slight rise in the level of THF and a compensatory fall in that of 10-formylTHF (Figure 6). An equally small effect on the folate pool was observed with LY231514, with a trend for THF and 10-formylTHF consistently opposite to that for raltitrexed. Based on an analogy to the observed accumulation of 10-formylTHF by LY309887 (Figure 5), the differential effects on 10-formylTHF and THF induced by LY231514 and raltitrexed (Figure 6) might be attributable to a difference in their effects on GARFT. This hypothesis, however, would have to be confirmed by direct measurement of metabolic flux through the de novo purine nucleotide biosynthetic pathway.

The major message from the folate analysis was the lack of DHF accumulation induced by either raltitrexed or LY231514 in CCRF-CEM cells under very similar conditions in which DHF was detected with methotrexate. When TS was strongly inhibited, there would be little or no conversion of 5,10-methyleneTHF to DHF, and DHFR would be effectively inactive. Thus, the lack of intracellular accumulation of DHF by LY231514 does not contradict the fact that this drug inhibits purified DHFR in vitro. However, it does suggest the primary target of LY231514 to be TS in CCRF-CEM cells. In the presence of significant TS inhibition, it is difficult to study the metabolic effect of a drug on DHFR. Concurrent inhibition of two sequential steps in the same metabolic pathway can be redundant with respect to the end product.

The intracellular level of each nucleotide is a balance of synthesis, use and salvage (Kornberg, 1980; Reichard, 1988). The biosynthetic pathways of individual nucleotides are interconnected in that they share several common intermediates, and that the end product of one pathway serves as an allosteric regulator of the enzymes in the others. Moreover, the ribonucleotides are the precursors for RNA, as well as for their respective deoxyribose counterparts, and the syntheses of deoxyribonucleotides and DNA are under cell cycle regulation.

Against this background is the well known fact that methotrexate exhibits inhibitory activities against the biosynthesis of both pyrimidine and purine nucleotides (Ackland and Schilsky, 1987). Thus, the depletion of both ATP and GTP caused by methotrexate observed here and by others (Kinahan et al, 1979; Taylor et al, 1982*a* and *b*) is consistent with the antipurine effect of methotrexate. Furthermore, similar depletion of ATP and GTP was observed with the GARFT inhibitor LY309887 and was reported for lometrexol (Pizzorno et al, 1991; Chong and Tattersall, 1995), as well as for other antipurine compounds, such as 6-methylmercaptopurine ribonucleoside (Cohen and Sadee, 1983). Our data for methotrexate and LY309887 implied that, if there was an antipurine effect, it should have been evident in the ribonucleotide pools. Thus, it was not surprising that raltitrexed, having no known antipurine effect, did not show any significant effects on ribonucleoside triphosphate levels. Similar results were obtained with LY231514, despite previous reports on its inhibition on purified GARFT ($K_i = 65$ nM for LY231514-glu$_5$) (Shih et al, 1997) in vitro. In the presence of possible salvage and other compensatory pathways, the magnitude of the anti-GARFT effect of LY231514 might be insufficient to cause significant

© Cancer Research Campaign 1998

impact on the intracellular ribonucleoside triphosphate pools in CCRF-CEM cells.

Depletion of intracellular dTTP is the most notable consequence of a retardation of the thymidylate cycle, involving inhibition of either TS or DHFR (Kinahan et al, 1979; Jackson, 1992; Aherne et al, 1996; Kunz, 1996; Weber et al, 1996). The fall of dTTP observed with methotrexate, raltitrexed and LY231514 was very rapid. On the other hand, although LY309887 also depleted dTTP, it did so much more gradually.

It is well known that when cells are nutritionally deprived of thymine, the intracellular level of dTTP falls together with simultaneous perturbations in the other three deoxyribonucleoside triphosphates (Kunz, 1996). For example, studies with 5-fluorodeoxycytidine and CB3717 have shown a rise in dATP level and a fall in dGTP level accompanying dTTP depletion (Newman and Santi, 1982; Jackson et al, 1983; Kwok and Tattersall, 1992). With raltitrexed and LY231514, we observed an accumulation of dATP together with the disappearance of both dCTP and dGTP, concomitant with dTTP depletion. The changes in dCTP and dGTP may be rationalised by what is known about dCMP deaminase and ribonucleoside diphosphate reductase, the two enzymes that control the biosynthesis of deoxyribonucleotide (Kinahan et al, 1979; Kornberg, 1980; Oliva et al, 1996). It is known that dCMP deaminase is inhibitable by dTTP. Possibly, the fall of dTTP as a result of TS inhibition could restore dCMP deaminase activity, causing dCMP to be converted to dUMP. This could in turn lead to dCTP depletion via the rapid intracellular equilibrium between the mono-, di- and triphosphate forms of the nucleoside. At the same time, ribonucleoside diphosphate reductase, which converts GDP to dGDP, is allosterically enhanced by dTTP and inhibited by dATP (Reichard, 1988). Thus, the fall of dGTP observed with reltitrexed and LY231514 here may be consistent with the retardation in the synthesis of dGDP under conditions of low dTTP and high dATP.

The mechanism for the changes in dATP levels is not well understood. However, it is interesting to note that for methotrexate, a drug with both antipyrimidine and antipurine effects, depletion of dTTP occurred without a concommitant increase in dATP. Furthermore, Chong and Tattersoll (1995) reported that the combination of a GARFT inhibitor and a TS inhibitor prevented the rise in the dATP pool seen with the TS inhibitor alone. Based on these observations, the difference in the rate of dATP accumulation induced by raltitrexed and LY231514 would be attributable to the difference in antipurine effect of the two drugs, a hypothesis that can be verified by direct evaluation of the metabolic flux from glycine to inosinic acid.

In conclusion, our study showed that the new folate analogue LY231514 was an antimetabolite distinct from methotrexate, LY309887 and raltitrexed. The data presented above are consistent with LY231514 in affecting the thymidylate cycle as well as possibly de novo purine nucleotide biosynthesis in CCRF-CEM cells. Although, in the present study with CCRF-CEM cells, a significant perturbation of the ribonucleotide pools was not observed with LY231514, comparing the changes in dATP induced with other folate-based drugs provided evidence suggestive of the possible presence of an effect on purine nucleotides. The inhibition of the thymidylate cycle contributed by strong inhibition of TS could mask the effect of LY231514 on DHFR, as a result of the two enzymes being sequential steps in the same metabolic pathway. As such, it will be interesting to study a cell line with overexpressed TS levels in future. Evaluation in other cell lines is in progress to confirm the generality of the observations reported in this paper. The phase I evaluation of LY231514 has been completed (Rinaldi et al, 1995, 1996; McDonald et al, 1996). A broad phase II clinical study using the q21 days at 500–600 mg $m^{-2}$ is currently in progress and preliminary results indicate early favourable responses in several tumour types (Clarke et al, 1997; Cripps et al, 1997; John et al, 1997; Miller et al, 1997; Rusthoven et al, 1997; Smith et al, 1997). It will be very interesting to see how the mechanistic subtleties between the folate-based drugs discussed in this paper eventually translate into differences in clinical efficacy and toxicity.

### ACKNOWLEDGEMENTS

We are extremely grateful to Dr Donald W Horne for the excellent guidance on folate pool analyses, to him as well as to Dr Dean Appling for critical reading of this manuscript, to Dr Richard G Moran and Dr Carmen J Allegra for very helpful discussions.

### REFERENCES

Ackland SP and Schilsky RL (1987) High-dose methotrexate: a critical reappraisal. *J Clin Oncol* **5**: 2017–2031

Aherne GW, Hardcastle A, Raynaud F and Jackman AL (1996) Immunoreactive dUMP and TTP pools as an index of thymidylate synthase inhibition; effect of Tomudex (ZD1694) and a nonpolyglutamated quinazoline antifolate (CB30900) in L1210 mouse leukaemia cells. *Biochem Pharmacol* **51**: 1293–1301

Allegra CJ, Fine RL, Drake JC and Chabner BA (1986) The effect of methotrexate on intracelllular folate pools in human MCF-7 breast cancer cells: evidence for direct inhibition of purine synthesis. *J Biol Chem* **261**: 6478–6485

Baram J, Allegra CJ, Fine RL and Chabner BA (1987) Effect of methotrexate on intracellular folate pools in purified myeloid precursor cells from normal human bone marrow. *J Clin Invest* **79**: 692–697

Benjamini Y and Hochberg Y (1995) Controlling the false discovery rate: a practical and powerful approach to multiple testing. *J R Statist Soc B* **57**: 289–300

Chong L and Tattersall MHN (1995) 5,10-dideazatetrahydrofolic acid reduces toxicity and deoxyadenosine triphosphate pool expansion in cultured L1210 cells treated with inhibitors of thymidylate synthase. *Biochem Pharmacol* **49**: 819–827

Clarke S, Boyer M, Millward M, Findlay M, Ackland S, Childs A, Brew S, Walcher V, Watt D and Campbell L (1997) Phase II study of LY231514 in patients with advanced non-small cell lung cancer (NSCLC). *Am Soc Clin Oncol* **16**: abstract 1670

Cohen MB and Sadee W (1983) Contribution of the depletions of guanine and adenine nucleotides to the cytotoxicity of purine starvation in the mouse T lymphoma cell line. *Cancer Res* **43**: 1587–1591

Cripps MC, Burnell M, Jolivet J, Lofters W, Fisher B, Panasci L, Iglesias J and Eisenhauer E (1997) Phase II study of a multi-targeted antifolate (LY231514) (MTA) as first-line therapy in patients with locally advanced or metastatic colorectal cancer (MCC). *Am Soc Clin Oncol* **16**: abstract 949

Garrett C and Santi DV (1979) A rapid and sensitive high pressure liquid chromatography assay for deoxyribonucleotide triphosphates in cell extracts. *Anal Biochem* **99**: 268–273

Habeck LL, Leitner TA, Shackelford KA, Gossett LS, Schultz RM, Andis SL, Shih C, Grindey GB and Mendelsohn LG (1994) A novel class of monoglutamated antifolates exhibits tight-binding inhibition of human glycinamide ribonucleotide formyltransferase and potent activity against solid tumours. *Cancer Res* **54**: 1021–1026

Habeck LL, Shih C, Gossett LS, Leitner TA, Schultz RM, Andis SL, Moran RG and Mendelsohn LG (1995) Substrate specificity of mammalian folylpolyglutamate synthetase for 5,10-dideazatetrahydrofolate analogs. *Mol Pharmacol* **48**: 326–333

Hanauske A-R (1996) The development of new chemotherapeutic agents. *Anti-Cancer Drugs* **7** (suppl.2): 29–32

Jackman AL and Calvert AH (1995) Folate-based thymidylate synthase inhibitors as anticancer drugs. *Ann Oncol* **6**: 871–881

Jackson RC (1992) *The Theoretical Foundations of Cancer Chemotherapy introduced by Computer Models*. Academic Press: San Diego, pp. 299–341

Jackson RC, Jackman AL and Calvert AH (1983) Biochemical effects of a quinazoline inhibitor of thymidylate synthase, N-(4-(N-((2-amino-4-hydroxy-6-quinazolinyl)methyl)prop-2-ynylamino)benzoyl)-L-glutamic acid (CB3717), on human lymphoblastoid cells. *Biochem Pharmacol* **32**: 3783–3790

John W, Clark J, Burris H, Picus J, Schulman L, Thornton D and Lochrer P (1997) A phase II trial of LY231514 in patients with metastatic colorectal cancer. *Am Soc Clin Oncol* **16**: abstract 1038

Kinahan JJ, Otten M and Grindey GB (1979) Evaluation of ribonucleoside and deoxyribonucleoside triphosphate pools in cultured leukemia cells during exposure to methotrexate or methotrexate plus thymidine. *Cancer Res* **39**: 3531–3539

Kornberg A (1980) *DNA Replication*. WH Freeman: San Francisco

Kunz BA (1996) Inhibitors of thymine nucleotide biosynthesis: antimetabolites that provoke genetic change via primary non-DNA targets. *Mutation Res* **355**: 129–140

Kwok JBJ and Tattersall MHN (1992) DNA fragmentation, dATP pool elevation and potentiation of antifolate cytotoxicity in L1210 cells by hypoxanthine. *Br J Cancer* **65**: 503–508

Matherly LH and Muench SP (1990) Evidence for a localized conversion of endogenous tetrahydrofolate cofactors to dihydrofolate as an important element in antifolate action in murine leukemia cells. *Biochem Pharmacol* **39**: 2005–2014

McDonald AC, Vasey PA, Walling J, Woodworth JR, Abrahams T, Bailey NP, Siddiqui N, Lind MJ, Cassidy J and Twelves CJ (1996) Phase 1 and pharmacokinetic study of LY231514, the multi-targeted antifolate, administered by daily × 5, q21 schedule. *Ann Oncol* **7** (suppl. 5): 608P

Mendelsohn LG, Shih C, Schultz RM and Worzalla JF (1996) Biochemistry and pharmacology of glycinamide ribonucleotide formyltransferase inhibitor: LY309887 and lometrexol. *Invest New Drugs* **14**: 287–294

Miller KD, Loehrer PJ, Picus J, Blanke C, John J, Shulman L, Burris H and Thornton D (1997) A phase II trial of LY231514 in patients with unresectable pancreatic cancer. *Am Soc Clin Oncol* **16**: abstract 1060

Morrison PF and Allegra CJ (1989) Folate cycle kinetics in human breast cancer cells. *J Biol Chem* **264**: 10552–10566

Newman EM and Santi DV (1982) Metabolism and mechanism of action of 5-fluorodeoxycytidine. *Proc Natl Acad Sci USA* **79**: 6419–6423

Oliva FJ, Collins MKL and Lopez-Rivas A (1996) dNTP pools imbalance as a signal to initiate apoptosis. *Experientia* **52**: 995–1000

Pizzorno G, Moroson BA, Cashmore AR and Beardsley GP (1991) (6R)-5,10-dideaza-5,6,7,8-tetrahydrofolic acid effects on nucleotide metabolism in CCRF-CEM human T-lymphoblast leukemia cells. *Cancer Res* **51**: 2291–2295

Reichard P (1988) Interaction between deoxyribonucleotide and DNA synthesis. *Annu Rev Biochem* **57**: 349–374

Rinaldi DA, Burris HA, Dorr FA, Woodworth JR, Kuhn JG, Eckardt JR, Rodriguez G, Corso SW, Fields SM, Langley C, Clark G, Faries D, Lu P and Von Hoff DD (1995) Initial phase I evaluation of the novel thymidylate synthase inhibitor, LY231514, using the modified continual reassessment method for dose escalation. *J Clin Oncol* **13**: 2842–2850

Rinaldi DA, Burris HA, Dorr FA, Rodriguez G, Eckardt SG, Fields SM, Woodworth JR, Kuhn JG, C. L, Clark G, Lu P and Von Hoff DD (1996) A phase I evaluation of LY231514, a multitargeted antifolate, administered every 21 days. *Proc ASCO* **15**: 1559

Rusthoven J, Eisenhauer E, Butts C, Gregg R, Dancey J, Fisher B and Iglesias J (1997) A phase II study of the multi-targeted antifolate LY231514 in patients with advanced non-small cell lung cancer. *Am Soc Clin Oncol* **16**: abstract 1728

Schultz RM (1995) Newer antifolates in cancer therapy. In *Progress in Drug Research*, **44**, Jucker E. (ed.), pp. 129–157, Birkhauser: Basle

Shih C, Chen VJ, Gossett LS, Gates SB, Mackellar WC, Habeck LL, Shackelford KA, Mendelsohn LG, Soose DJ, Patel VF, Andis SL, Bewley JR, Rayl EA, Moroson BA, Bearsley GP, Kohler W, Ratnam M and Schultz RM (1997) LY231514, a pyrrolo[2,3-d]pyrimidine-based antifolate that inhibits multiple folate-requiring enzymes. *Cancer Res* **57**: 1116–1123

Smith IE, Miles DW, Coleman RE, Lind MJ, McCarthy S and Chick J (1997) Phase II study of LY231514 (MTA) in patients (pts) with locally recurrent or metastatic breast cancer (LR/MBC) – an interim report. *Am Soc Clin Oncol* **16**: abstract 671

Strong WB, Tendler SJ, Seither RL, Goldman D and Schirch V (1990) Purification and properties of serine methyltransferase and C1-tetrahydrofolate synthase from L1210 cells. *J Biol Chem* **265**: 12149–12155

Taylor EC, Kuhnt D, Shih C, Rinzel SM, Grindey GB, Barredo L, Jannatipour M and Moran RG (1992) A dideazatetrahydrofolate analogue lacking a chiral center at C-6, N-[4-[2-(2-amino-3.4-dihydro-4-oxo-7H-pyrrolo[2,3-d]pyrimidin-5-y1(ethyl]benzoyl]-L-glutamic acid, is an inhibitor of thymidylate synthase. *J Med Chem* **35**: 4450–4454

Taylor IW, Slowiaczek P, Francis PR and Tattersall MHN (1982*a*) Purine modulation of methotrexate cytotoxicity in mammalian cell lines. *Cancer Res* **42**: 5159–5164

Taylor IW, Slowiaczek P, Francis PR and Tattersall MHN (1982*b*) Biochemical and cell cycle perturbations in methotrexate-treated cells. *Mol Pharmacol* **21**: 204–210

Trent DF, Seither RL and Goldman ID (1991) Compartmentation of intracellular folates. *Biochem Pharmacol* **42**: 1015–1019

Webber S, Bartlett CA, Boritzki TJ, Hilliard JA, Howland EF, Johnston AL, Kosa M, Margosiak SA, Morse CA and Shetty BV (1996) AG337, a novel lipophilic thymidylate synthase inhibitor: *in vitro* and *in vivo* preclinical studies. *Cancer Chemother Pharmacol* **37**: 509–517

Wilson SD and Horne DW (1983) Evaluation of ascorbic acid in protecting labile folic acid derivatives. *Proc Natl Acad Sci USA* **80**: 6500–6504

 © Cancer Research Campaign 1998